# Immunotherapy for Melanoma in Patients with Altered Immune Systems: Unique Challenges and Clinical Considerations

**DOI:** 10.1007/s12325-025-03453-8

**Published:** 2025-12-29

**Authors:** Bryan L. Peacker, Jonathan C. Hwang, Rebecca I. Hartman

**Affiliations:** 1 Harvard Medical School, Boston, MA USA; 2 University of Pittsburgh School of Medicine, Pittsburgh, PA USA; 3 Department of Dermatology, Brigham and Women’s Hospital, Boston, MA USA; 4 Dermatology Section, VA Boston Healthcare System, Jamaica Plain, MA USA

**Keywords:** Melanoma treatment, Immune checkpoint inhibitors, Immunotherapy, Altered immune systems, Special populations, HIV, Tuberculosis, Transplantation, Pregnancy, Autoimmune diseases

## Abstract

While immunotherapy has been widely adopted for the treatment of melanoma, its application in patients with complex comorbidities remains challenging. This review explores evidence on the efficacy, safety, and special considerations for the use of immunotherapy in patients with altered immune systems, including patients with human immunodeficiency virus (HIV), tuberculosis, solid organ or hematopoietic cell transplantation, autoimmune diseases, and pregnant women. Despite data emphasizing the feasibility of immunotherapy treatment in these populations, standardized management algorithms are lacking. Future research should consider either including these patients in prospective trials or attempting to collect data via registries to provide more clarity on the management of immunologically vulnerable patients with melanoma.

## Key Summary Points

**Table:** 

Immunotherapy is effective for melanoma, including in patients with altered immune systems, but presents unique challenges.
Well-controlled human immunodeficiency virus (HIV) infection is not an absolute contraindication to immunotherapy.
Immunotherapy may elevate risk of *Mycobacterium tuberculosis* infection, making screening an important step prior to initiation of therapy.
Immunotherapy in transplant recipients and pregnant patients is not recommended because of concerns about the risk of adverse events, but limited data necessitate cautious, case-by-case evaluation.
Patients with preexisting autoimmune diseases can safely receive immunotherapy but are at increased risk of immune-related adverse events, with some cases requiring immunosuppressive management.
More research is needed to address gaps in safety and efficacy for these special patient populations with melanoma.

## Introduction

Immunotherapy has transformed conventional approaches to melanoma treatment. These therapies modulate the immune system to elicit a cytotoxic response in melanoma cells and have demonstrated pronounced efficacy in clinical trials [1]. A variety of approaches for advanced melanoma are available or in development, including immune checkpoint inhibitors (ICIs), oncolytic viruses, chimeric antigen receptor T cell (CAR-T) therapy, tumor-infiltrating lymphocyte (TIL) therapy, and cancer vaccines. At present, no CAR-T therapies or cancer vaccines are approved for melanoma, and only one TIL therapy has received approval. Data on non-ICI immunotherapies are limited [2].

As of this writing, five ICIs are approved for melanoma, along with one oncolytic virus and one TIL therapy (Table 1). The approved ICIs are pembrolizumab and nivolumab (anti-PD-1 antibodies), atezolizumab (anti-PD-L1 antibody), ipilimumab (anti-CTLA-4 antibody), and relatlimab (anti-LAG-3 antibody) [3]. Relatlimab is approved only in combination with nivolumab, while atezolizumab is approved in combination with cobimetinib (MEK inhibitor) and vemurafenib (BRAF inhibitor) for BRAF V600-positive melanoma [3]. Talimogene laherparepvec (T-VEC), the only approved oncolytic virus therapy, and lifileucel, a TIL therapy, are approved for patients with unresectable melanoma [3]. Trial data that supported the approval of these agents are summarized in Table 1.

**Table 1:** Summary of key clinical trial data used to support the approval of immunotherapies for metastatic or unresectable melanoma

Study	Treatment arms	Objective response rate^a^	Grade 3 or greater AEs^a^
Hodi et al. 2010 (MDX010-20) [87]	Ipilimumab + gp100 vs. ipilimumab vs. gp100	5.7% (*n* = 403) vs. 10.9% (*n* = 137) vs. 1.5% (*n* = 136)	10.2% (*n* = 380) vs. 14.5% (*n* = 131) vs. 3.0% (*n* = 132)
Robert et al. 2015 (CheckMate 066) [88]	Nivolumab vs. dacarbazine	40% (*n* = 209) vs. 13.9% (*n* = 209)	11.7% (*n* = 209) vs. 17.6% (*n* = 209)
Robert et al. 2015 (KEYNOTE-006) [89]	Pembrolizumab every 2 weeks vs. pembrolizumab every 3 weeks vs. ipilimumab every 3 weeks	33.7% (*n* = 278) vs. 32.9% (*n* = 278) vs. 11.9% (*n* = 278)	13.3% (*n* = 278) vs. 10.1% (*n* = 278) vs. 19.9% (*n* = 278)
Wolchok et al. 2017 (CheckMate 067) [90]	Nivolumab plus ipilimumab vs. nivolumab vs. ipilimumab	57.6% (*n* = 314) vs. 43.7% (*n* = 316) vs. 19.0% (*n* = 315)	55.0% (*n* = 314) vs. 16.3% (*n* = 316) vs. 27.3% (*n* = 315)
Gutzmer et al. 2020 (IMspire150) [91]	Atezolizumab + vemurafenib + cobimetinib vs. placebo + vemurafenib + cobimetinib	66.3% (*n* = 256) vs. 65.0% (*n* = 258)	79% (*n* = 230) vs. 73% (*n* = 281)
Tawbi et al. 2022 [92]; Long et al. 2023 [93] (RELATIVITY-047)^b^	Relatlimab + nivolumab vs. nivolumab	43.1% (*n* = 355) vs. 32.6% (*n* = 359)	18.9% (*n* = 355) vs. 9.7% (*n* = 359)
Andtbacka et al. 2015 (OPTiM) [94]	Talimogene laherparepvec vs. granulocyte–macrophage colony-stimulating factor	26.4% (*n* = 295) vs. 5.7% (*n* = 141)	11% (*n* = 292) vs. 5% (*n* = 127)
Chesney et al. 2022 (MASTERKEY-265) [95]	Talimogene laherparepvec + pembrolizumab vs. placebo + pembrolizumab	48.6% (*n* = 346) vs. 41.3% (*n* = 346)	20.7% (*n* = 345) vs. 19.5% (*n* = 343)
Chesney et al. 2022 (C-144–01) [96]	Lifileucel (single arm)	31.4% (*n* = 153)	76.9% (*n* = 153)

^a^Patient numbers reflect the total number of patients in each arm

^b^Response rate data from the RELATIVITY-047 trial was reported in Long et al. 2023 [93], while safety data was reported in Tawbi et al. 2022 [92]

While many clinical guidelines include immunotherapy in treatment decision algorithms, there is little guidance on certain patient populations with altered immune systems, including those with human immunodeficiency virus (HIV), transplant recipients, and pregnant women. This review synthesizes evidence on immunotherapy in these populations, focusing on ICI safety, efficacy, and challenges in diverse immune contexts.

## Methods

We conducted a search of PubMed from January 1, 2010 to July 31, 2025, for English-language articles using the following query: (“melanoma”) AND (“immune checkpoint inhibitors” OR “oncolytic viruses” OR “CAR-T therapy” OR “chimeric antigen receptor T-cell” OR “tumor-infiltrating lymphocyte therapy” OR “TIL therapy” OR “cancer vaccines”) AND (“transplant recipients” OR “pregnancy” OR “pregnant women” OR “pregnant patient*” OR “HIV” OR “human immunodeficiency virus” OR “autoimmune diseases” OR “autoimmune disorder” OR “second primary” OR “multiple malignant*”). We considered articles that reported case–control studies, case series, cohort studies, clinical trials, and clinical guidelines related to the patient populations in our search. Conference proceedings and abstracts were excluded.

## Results

### Patients with HIV

Despite widespread antiretroviral therapy (ART), patients with HIV face an increased risk of melanoma [4]. While concern about immune system dysfunction in patients with HIV has led to their exclusion from many clinical trials for immunotherapy, strong evidence indicates that well-controlled HIV is not a contraindication to ICIs [5–7]. The DURVAST study, a phase 2 trial in patients with HIV and solid tumors, found that treatment with durvalumab was feasible and not associated with severe toxicity or decreases in CD4^+^ T cell counts [8]. In a larger prospective observational study of 150 patients with HIV, grade ≥ 3 immune-related adverse events (irAEs) occurred in 15.0%, without worsening viremia or immunological parameters [9]. Similarly, a systematic review of 73 patients with HIV treated with ICIs reported grade ≥ 3 irAEs in 8.6% of patients, comparable to that of the general cancer population [10]. In light of this evidence, the Society for Immunotherapy for Cancer (SITC) guidelines for melanoma recommend initiating ART in patients with poorly controlled HIV with a goal CD^+^ T cell count ≥ 100/µL, HIV viral load < 200 copies/mL, and treatment with ART for at least 4 weeks before starting ICIs [11]. Well-controlled HIV infection alone should not preclude ICI therapy.

### Transplant Recipients

Patients receiving immunosuppression to maintain graft tolerance after solid organ or allogeneic hematopoietic cell transplantation (HCT) are at significantly increased risk of developing malignancies, including melanoma [12]. For transplant patients with melanoma in whom immunotherapy would otherwise be indicated, graft rejection is a major concern, as is potential blunting of antitumor response due to immunosuppression. Studies have not found differing rejection rates between different ICI classes [13–16]. In a systematic review of 31 organ transplant recipients treated with ICIs for melanoma, eight (25%) experienced graft rejection, and overall response rates (ORRs) were similar to those reported in clinical trials [16]. A single-institution retrospective study of 39 transplant recipients, including 24 with melanoma, similarly found that 31% of patients permanently discontinued ICIs as a result of graft rejection; among these patients, mortality was 46% [15]. A meta-analysis of 90 patients treated with ICI for cutaneous malignancies found graft rejection rates of 41%, with an ORR for melanoma of 31%. There were no significant differences in graft rejection among kidney, liver, or heart transplants, suggesting comparable outcomes across transplant organ type [17].

Data on optimal timing of transplantation after immunotherapy are even more sparse. No studies have examined the feasibility of transplantation after immunotherapy for melanoma, though it has been reported for other malignancies. In Hodgkin lymphoma, a longer interval between anti-PD-1 therapy and HCT was associated with a lower risk of severe acute graft-versus-host disease (GVHD), though rates of graft rejection were not reported [18]. In a retrospective cohort of patients with hepatocellular carcinoma who received ICIs prior to liver transplantation, ICI washout periods less than 30 days were associated with 21.3 times higher odds of graft rejection compared with washout periods more than 50 days; a 30–50-day washout had 9.48 times higher odds of rejection [19]. Across several studies, patients who received ICIs before HCT had higher rates of irAEs [20].

Patients receiving HCT are unique compared to solid organ transplant recipients (SOTRs) since the graft-versus-tumor effect is often desirable in the former, and immunotherapy may potentiate this effect. There are three reported cases of HCT recipients who developed melanoma after transplant and were treated with pembrolizumab; all achieved partial responses without GVHD or graft rejection [21, 22]. In contrast, larger studies investigating the treatment of other cancers have suggested an increased risk of GVHD with ICIs [23]. In a phase 1 multicenter study of high-dose ipilimumab for relapsed immunologic cancer, seven of 22 patients (32%) had a response, and dose-limiting GVHD occurred in four (18%) patients [24, 25].

Only one clinical guideline, from the Advisory Committee of the Spanish Melanoma Group (GEM), includes recommendations for transplant recipients. GEM states that ICIs are not absolutely contraindicated in SOTRs, but that physicians should consider alternative therapies, monitor immunosuppression closely with periodic examinations, and hold ICIs during graft rejection, with possible reinitiation after recovery [26]. Renal transplant recipients may receive dialysis in the event of graft failure, suggesting that the risk–benefit calculation may differ by organ type. However, a return to dialysis is often considered an unacceptable outcome that imposes a serious long-term burden on quality of life. For HCT recipients, GEM recommends considering ICIs only when there are no other treatment options, as a result of the high risk of GVHD and irAEs [26].

Other forms of immunotherapy, such as TIL or cancer vaccines, are not discussed by GEM [26]. TIL therapy has shown promise in melanoma treatment, with ORRs of 36% observed in a phase 2 open-label, single-arm, multicenter clinical trial [27]. Recent 2024 expert consensus guidelines on TIL therapy recommend excluding patients who require immunosuppressive doses of systemic steroids or biological agents [28].

### Tuberculosis

Tuberculosis is the leading cause of death from an infectious agent worldwide [29]. While immunotherapy does not directly cause immunosuppression, treatment of irAEs with immunosuppressants may increase the risk of tuberculosis. Even without immunosuppression, ICIs may paradoxically reactivate latent tuberculosis infection (LTBI), possibly through increased Th1 cell-mediated activity that disrupts bacterial control [30, 31]. Excessive immune responses to antigens may also reactivate or worsen tuberculosis through failed tolerance, though this mechanism is still unknown [32, 33]. Notably, no cases of tuberculosis have been reported with anti-CTLA-4 monotherapy, though two cases have been described with combined anti-CTLA-4 and anti-PD-1 therapy [34, 35].

The risk of active tuberculosis is higher in patients with any malignancy [36], and multiple studies have shown an association between ICIs and tuberculosis infection or reactivation, including in melanoma [30, 37–39]. A recent systematic review and meta-analysis of patients treated with anti-PD-1 or anti-PD-L1 blockade reported a pooled incidence of 2000 cases per 100,000, which is 35 times higher than the general population [40]. An analysis of South Korea insurance claims data showed an eightfold higher incidence of tuberculosis in patients treated with ICIs, although multivariable analyses did not confirm a significant difference in the hazard of developing tuberculosis [41]. The study used the cancer diagnosis rather than ICI initiation as the index date, potentially introducing immortal time bias. A prospective study including Japanese patients with lung cancer found tuberculosis seroconversion in 3.3% (4/123) of patients during ICI treatment [42]. Similarly, a retrospective study from Taiwan found an increased risk of LTBI or reactivation in patients with lung cancer treated with ICIs compared to tyrosine kinase inhibitors [43]. In this study, seroconversion served as a proxy for latent or reactivated tuberculosis, but no comparator group was available. No studies have adjusted for chemotherapy, corticosteroids, or other immunosuppressive medications, which are important confounders, particularly in light of existing data suggesting high-dose corticosteroids are associated with worse survival outcomes when used to treat irAEs in patients with melanoma [44, 45]. Nevertheless, current evidence supports a probable association between both cancer and ICIs with tuberculosis.

The most recent US Preventive Services Task Force (USPSTF) recommendations do not address tuberculosis screening in patients receiving immunotherapy [46]. The SITC guidelines on irAEs recommend screening only for patients considered for anti-tumor necrosis factor (TNF) therapy [47], while a 2016 collaborative position paper from France suggested testing only in patients requiring additional immunosuppression in the setting of severe toxicity [48]. The GEM Advisory Committee guidelines on the treatment of melanoma, however, recommend screening for tuberculosis prior to ICI administration, with further evaluation to rule out active tuberculosis in those who screen positive [26]. The GEM committee also advises biopsy of suspicious lung lesions [47]. In patients who screen positive for LTBI without evidence of active tuberculosis, tuberculosis chemoprophylaxis is generally not recommended, though 4 weeks of isoniazid and rifapentine [49] can be considered prior to immunotherapy only after weighing drug–drug interactions, hepatotoxicity, and the risk of delaying immunotherapy [26]. The GEM committee does not make a formal recommendation regarding chemoprophylaxis during immunotherapy, but recommends against delaying immunotherapy in patients with advanced cancer and states that chemoprophylaxis should be mainly considered in young patients with planned adjuvant treatment [26].

In cases of active tuberculosis or reactivation, ICI discontinuation and guideline-directed medical therapy is recommended by the GEM committee, given the potential for an exaggerated immune response [26, 50]. There is no consensus on ICI rechallenge timing after tuberculosis infection. Close monitoring for hepatotoxicity is recommended, particularly for treatment with isoniazid [26]. Some reports discontinued ICI treatment in patients with active tuberculosis, while others reported successful treatment of tuberculosis with antitubercular medications while continuing ICI treatment [51–53], indicating that concomitant treatment may be feasible without an amplified immune reaction.

### Diagnosis and Treatment During Pregnancy

Melanoma is one of the most common malignancies diagnosed during pregnancy, accounting for up to one-third of cancer cases in some studies [54]. Pregnancy-associated melanoma (PAM) is linked to increased peritumoral lymphangiogenesis and higher rates of placental and fetal metastases (reported in 22% and 59% of cases, respectively) [55, 56]. Maternal mortality is estimated to be 17–56% higher compared to nonpregnant patients, and fetal metastases carry a one-year survival of 51% [56, 57].

The recent incidence of fetal metastases has been demonstrated to be lower than previously expected. A 2023 multicenter study showed that among 67 pregnancies in women with stage III/IV melanoma, none resulted in metastasis to the placenta or the fetus [58]. Placental screening and close observation of children born to mothers with antenatal advanced melanoma are essential due to the grave consequences of vertical transmission [58].

Less is known about the safety and efficacy of immunotherapy during pregnancy. ICIs are known to cross the placenta, particularly in the second and third trimesters of gestation, and have been linked to irAEs in the fetus. A study using the World Health Organization international pharmacovigilance database, VigiBase, of 91 patients with pregnancy-associated cancer who received ICIs in the antenatal period found 45 adverse outcomes (49%), of which 3 (3.2%) were possibly immune-related maternofetal events. However, there was no overrepresentation in adverse maternal, fetal, or neonatal outcomes compared to other anticancer therapies. The exception was preterm birth, which was significantly more common with combination anti-PD1 and anti-CTLA4 therapy compared to other anticancer drugs [59]. A separate case series of seven pregnancies with nine neonates found complications in five (71%) of pregnancies [60]. Though rare irAEs or pregnancy loss are possible, ICI treatment during pregnancy is not absolutely contraindicated [26], and any use should be considered on a case-by-case basis.

### Autoimmune Diseases

Patients with preexisting autoimmune disease have historically been excluded from immunotherapy trials for melanoma due to concerns of disease flares or severe irAEs. Even in studies that examined risk of autoimmune disease development in patients receiving ICI, many did not distinguish chronic irAEs from acute or subacute irAEs, making it difficult to estimate de novo autoimmunity after immunotherapy in patients without baseline autoimmune disease. There is ongoing debate regarding whether baseline use of immunosuppressive agents when initiating ICIs may attenuate efficacy [61]. It is also unclear whether specific autoimmune disease phenotypes or severity influence the risk of irAEs or alter therapeutic response. The clinical trials instrumental for the approval of the first ICI therapies for melanoma excluded those with autoimmune disease [62]. In fact, 55% of patients with metastatic melanoma in a nationwide cohort would not have met criteria for inclusion in the phase III trials that preceded ICI approvals, limiting generalizability [63]. Data for ICI safety and effectiveness for metastatic melanoma in those with preexisting autoimmune disease are limited to retrospective studies and case series.

Autoimmune disease is common among patients with metastatic melanoma. In a cohort of more than 12,000 patients diagnosed with melanoma between 2004 and 2014, autoimmune disease prevalence increased from 17.1% to 28.3%, outpacing the general population (7.9% to 9.2%) during the same period [64]. Transcriptomic data have also linked melanoma and autoimmunity, with differential expression of several genes implicated in autoimmune dysfunction in melanoma tissue compared with normal skin [65].

Multiple retrospective studies indicate that ICI treatment in patients with autoimmune disease can achieve response rates and survival outcomes similar to those without autoimmune disease; some studies have even suggested improved overall survival in patients with melanoma with baseline autoimmune disease [66, 67]. Other studies have demonstrated that irAEs are associated with improved outcomes in patients with melanoma, suggesting irAEs may be a clinical indicator of ICI activity [68]. Rates of irAEs and flares, however, vary by autoimmune disease type [69–72]. In a multicenter German series of 41 patients with autoimmune disease treated with ipilimumab, 29% developed new irAEs and 12% achieved a response [69]. Johnson et al. tracked 30 patients with preexisting autoimmune disease who were given ipilimumab treatment. Half of the patients had neither autoimmune disease flares nor irAEs; 27% experienced autoimmune disease flares that were managed with corticosteroids, and 10 (33%) patients experienced grade 3–5 irAEs, also reversible with corticosteroids (infliximab in two cases). Six (20%) patients experienced an objective response [73].

The above findings parallel outcomes seen with other ICI regimens. A study following preexisting autoimmune diseases and/or major irAEs treated with anti-PD-1 observed a 33% ORR. Immunotoxicities were frequent but often mild and easily managed [74]. In a nationwide cohort, van der Kooij et al. examined rates of irAEs in 3952 patients without autoimmune disease and compared them to rates in 415 patients with autoimmune disease. They found similar ORR and survival in patients with and without preexisting autoimmune disease: ORR was 10% vs. 16% with anti-CTLA-4, 40% vs. 44% with anti-PD-1, and 39% vs. 43% with combination therapy. Rates of grade ≥ 3 irAEs were also comparable: 30% vs. 30% with anti-CTLA-4, 17% vs. 13% with anti-PD-1, and 44% vs. 48% with combination therapy. However, certain subgroups had elevated irAE risk. Patients with inflammatory bowel disease had higher rates of colitis induced by anti-PD-1 (19% vs. 3%) and more frequent treatment discontinuation (17% vs. 9%) [70]. A subsequent commentary noted that inclusion of milder autoimmune conditions, such as thyroid disease, may have underestimated irAE burden and that the absence of reported flare data limits interpretation [70]. These observations are consistent with a multicenter retrospective study of 112 patients with preexisting autoimmune disease receiving ICIs, in which 71% experienced a flare or new irAE. Flares occurred in 47%, and 43% required immunosuppressive therapy; however, permanent discontinuation of ICI was needed in only 21%, and most toxicities were reported as manageable [71]. In a multicenter study of 55 patients with autoimmune disease and advanced melanoma treated with a combination of ipilimumab and anti-PD-1, 33% had autoimmune disease flares, 67% developed unrelated irAEs, and 36% discontinued ICIs as a result of toxicity. ORR was 55%, with 77% of responses ongoing. Patients on baseline immunosuppression had higher flare risk and shorter overall survival [75]. The above findings suggest that some ICIs can be used in select patients with autoimmune disease, with manageable toxicity and outcomes within the range of general population estimates. However, risks vary by autoimmune disease subtype, and prospective data are limited for more recently approved ICIs. American Society of Clinical Oncology (ASCO) clinical guidelines recommend using selective immunosuppressants for 2–4 weeks prior to ICI treatment, followed by continued selective immunosuppression during therapy [76].

Recent studies have studied patients with autoimmune disease and concomitant advanced melanoma more in-depth. A phase Ib trial (AIM-NIVO, NCT03816345) is actively enrolling patients with specific autoimmune conditions and advanced cancers to prospectively evaluate the safety and efficacy of nivolumab, with disease-specific cohorts and biomarker exploration built into the study design [77]. Additional trials including patients with autoimmune disease are needed to better define treatment risks and outcomes in this population.

### Second Primary Malignancies

Patients with melanoma have an increased risk of a second primary malignancy [78], which is associated with worse prognosis [79]. No prospective immunotherapy trials have specifically enrolled patients with second primary malignancies. There are a limited number of case reports that suggest that a single ICI regimen can feasibly treat melanoma and a concurrent malignancy in patients with xeroderma pigmentosum [80, 81]. In patients with melanoma without genetic syndromes that predispose to cancer, some have reported successful treatment of both tumors [82, 83], while others have not [84, 85]. More studies are necessary to elucidate whether patients with melanoma and another primary malignancy are more likely to experience irAEs.

## Discussion

Immunotherapy represents a breakthrough in melanoma treatment and may yield favorable results even in patient populations with altered immune systems (Table 2). In this review, we provide an overview of the unique challenges faced when treating these patients. Although the existing evidence base is growing, many questions remain unanswered. Biomarkers that can help predict patient response to immunotherapy may prove helpful when choosing treatment strategies. Counseling patients with melanoma and altered immune systems should center around individualized risk–benefit discussions grounded in available data. Table 3 provides a summary of considerations for the clinical management of these patients. Importantly, careful attention to institution- and country-specific regulations regarding off-label use is essential, since immunotherapy use in these patient populations is off-label.

**Table 2:** Objective response rates and grade ≥ 3 irAEs or GVHD in studies included in this review

Special population	Objective response rate (ORR)^a^ % (*n*)	Grade ≥ 3 irAEs or GVHD % (*n*)	References
Patients with HIV	11% (38)^b^–69% (13)^c^	8% (390)–14% (21)	El Zarif et al. 2023 [97] Shah et al. 2019 [6]
Latent or active tuberculosis	NR	NR	
SOTRs	27% (11)^d^–50% (20)^e^	NR	Abdel-Wahab et al. 2019 [15]
Allogeneic HCT	25% (16)–48% (123)^f^	25% (51)^f^–38% (8)^g^	Ijaz et al. 2019 [23] Shatila et al. 2025 [20] Apostolova et al. 2023 [25]
Pregnant women	NR; Median PFS 16.0 months (7)^h^	NR	Andrikopoulou et al. 2021 [60]
Pre‑existing autoimmune disease	30% (132)^i^–32% (282)	Flares: 7% (426) New-onset: 12% (299) ^i^	Van der Kooij et al. 2021 [70] Liu et al. 2024 [72]

*HIV* human immunodeficiency virus,* HCT* hematopoietic cell transplant,* NR* not reported,* irAEs* immune-related adverse events,* GVHD* graft-versus-host disease,* PFS* progression-free survival,* SOTR* solid organ transplant recipients

^a^Objective response rate is defined as the proportion of patients who achieved a partial or complete response to immunotherapy. Patient counts reflect the total number of treated patients unless otherwise specified

^b^Patients with head and neck cancers

^c^Patients with skin cancers

^d^Patients who had received a renal transplant prior to therapy

^e^Patients who had received a liver transplant prior to therapy

^f^Systematic review; patient counts reflect patients treated after allogeneic HCT among included studies that were evaluable for ORR or grade ≥ 3 GVHD

^g^Retrospective single-center study; patient counts reflect patients treated after allogeneic HCT

^h^Systematic review including seven pregnancies and nine neonates from case reports

^i^Systematic review and meta-analysis of nine studies; pooled ORR and grade 3–4 irAE incidence were calculated using a random effects model

**Table 3:** Summary of considerations for immunotherapy use in patient populations included in this review

Patient population	Considerations
HIV	Well-controlled HIV is not an absolute contraindication for immunotherapy treatment. However, consider requiring CD4^+^ T cell count ≥ 100/µL, HIV viral load < 200 copies/mL, and treatment with ART for at least 4 weeks prior to the start of immunotherapy [26]
Latent tuberculosis	Consider screening patients before immunotherapy. If positive for LTBI, treat with a preferred LTBI regimen before or concurrent with immunotherapy. Preferred regimens from the from the National Tuberculosis Controllers Association and CDC [98] are 3 months of once-weekly isoniazid plus rifapentine, 4 months of daily rifampin, or 3 months of daily isoniazid plus rifampin. Alternative regimens are 6 or 9 months of daily isoniazid. Monitor patients closely for hepatotoxicitity from isoniazid
Active tuberculosis	If active tuberculosis is diagnosed, withhold immunotherapy. Start multidrug therapy based on the ATS/CDC/ERS/IDSA Clinical Practice Guideline recommendations [50]: Adults with isoniazid/rifampin-susceptible TB: 4-month regimen of isoniazid, rifapentine, pyrazinamide, and moxifloxacin Children with nonsevere, isoniazid/rifampin-susceptible TB: 4-month regimen of isoniazid, rifampin, pyrazinamide, with or without ethambutol Rifampin/fluoroquinolone-resistant TB: 6-month bedaquiline, pretomanid, and linezolid (BPaL) regimen Rifampin-resistant, fluoroquinolone-susceptible TB: 6-month bedaquiline, pretomanid, linezolid, and moxifloxacin (BPaLM) regimen Immunotherapy may be restarted in on a case-by-case basis consultation with infectious diseases specialists
Organ transplantation	Immunotherapy may be considered if no other treatment options exist, but risks of graft rejection and GVHD must be carefully balanced. Organ-specific considerations (i.e., dialysis for renal transplants) should be carefully weighed in consultation with solid organ transplantation specialists
Hematopoietic cell transplantation	Immunotherapy may be considered if no other treatment options exist, but risks of graft rejection and GVHD must be carefully balanced in consultation with bone marrow transplantation specialists
Pregnancy	Avoid immunotherapy treatment during pregnancy unless benefits outweigh risks with close monitoring
Autoimmune disease	Consider replacing nonselective agents with selective immunosuppressants 2–4 weeks prior to immunotherapy initiation when disease stage allows. Monitor for irAEs

*ATS* American Thoracic Society, *BPaL* bedaquiline, pretomanid, and linezolid, *BPaLM* bedaquiline, pretomanid, linezolid, and moxifloxacin, *CDC* Centers for Disease Control and Prevention, *ERS* European Respiratory Society, *IDSA* Infectious Diseases Society of America, *HIV* human immunodeficiency virus, *ART* antiretroviral therapy, *LTBI* latent tuberculosis infection, *GVHD* graft-versus-host disease, *irAEs* immune-related adverse events

An essential limitation of the evidence described in this review is the reliance on retrospective studies and case reports, which are prone to biases and unmeasured confounders. Furthermore, while our search included forms of immunotherapy other than ICIs, few or no studies were identified for most of these patient populations, mainly due to the exclusion of these patients from clinical trials, and since Food and Drug Administration approvals were only recently granted. When possible, future studies should use validated terminology for treatment response and adverse events, such as the immune-modified Response Evaluation Criteria in Solid Tumors (imRECIST) and the Common Terminology Criteria for Adverse Events (CTCAE). Prior reports have noted a lack of standardization of immune-related adverse events, even among studies that were associated with regulatory approvals [86].

Even in the face of complex comorbidities, immunotherapy remains a key component of the treatment of melanoma. A multidisciplinary and tailored approach should underpin management in these patients. More robust data that includes these patient populations should continue to be generated to guide clinical practice and improve outcomes for all patients.

## Data Availability

Data sharing is not applicable to this article as no datasets were generated or analyzed during the current study.
